# Development of physical fitness in Austrian primary school children: A longitudinal study among overweight and non-overweight children over 2.5 years

**DOI:** 10.1007/s00508-018-1336-x

**Published:** 2018-04-17

**Authors:** Gerhard Ruedl, Dominik Franz, Anika Frühauf, Martin Kopp, Martin Niedermeier, Clemens Drenowatz, Klaus Greier

**Affiliations:** 1 Deptartment of Sport Science, University of Innsbruck, Fürstenweg 185, 6020 Innsbruck, Austria; 2 Division of Physical Education, University of Education Upper Austria, Linz, Austria; 3 Division of Physical Education, University College of Education (KPH) Stams, Stams, Austria

**Keywords:** School children, Overweight, Obesity, Motor performance, Childhood

## Abstract

**Background:**

Physical activity and physical fitness play an important role in the prevention of overweight and obesity in childhood and adolescence and reduce the risk of becoming overweight or obese in adulthood.

**Aim:**

To evaluate the development of physical fitness in overweight and non-overweight primary school children from the first to third grades.

**Methods:**

Using a longitudinal study design, body height and weight as well as physical fitness of primary school children from Tyrol, Austria were measured five times during a period of 2.5 years using the German motor performance test (DMT 6–18).

**Results:**

In total, 266 children (55% boys) with a mean age of 6.4 ± 0.5 years at baseline participated. The proportion of overweight children was 11% at baseline and 22% at the fifth time point. Overweight children showed a significantly lower physical fitness level (mean total z‑score of DMT6–18) at all 5 time points (Hedges g: 0.64–1.09). Repeated measurement analyses of variances showed a significant increase of physical fitness over time among overweight (partial η^2^: 0.12) and non-overweight (partial η^2^: 0.29) children. With respect to gender, physical fitness significantly increased over time among overweight (partial η^2^: 0.20) and non-overweight (partial η^2^: 0.28) girls, as well as among non-overweight boys (partial η^2^: 0.31) but not among overweight boys (partial η^2^: 0.07).

**Conclusion:**

Overweight and non-overweight primary school children significantly increased their physical fitness over the study period; however, overweight children showed a significantly lower physical fitness level at all test time points and did not even achieve the mean baseline fitness level of non-overweight children. With respect to the increasing percentage of overweight children over the study period, evidence-based preventive measures to reduce overweight and increase physical fitness should be implemented at the earliest in primary schools with a special focus on overweight boys.

## Introduction

The increasing prevalence of childhood overweight and obesity in past decades represents a major public health problem of the twenty-first century [1, 2]. There is evidence that overweight and obese children and adolescents have an increased risk of becoming overweight adults [3]. Even in the absence of adult obesity, childhood obesity has been shown to increase chronic disease risk [4]. Physical activity plays an important role in the prevention of overweight and obesity in childhood and adolescence and reduces the risk of becoming overweight or obese in adulthood [5]. Overall, there is evidence that overweight and obesity, physical inactivity and a lack of fitness at young age are associated with increasing prevalence of cardiovascular risk factors, orthopedic problems, and psychosocial constraints later on [6–8] leading to a reduced quality of life [9] as well as to a reduced life expectancy of overweight people by several years [10]. Unfortunately, a large proportion of children and adolescents do not meet the recommended physical activity guidelines as active behavior has been replaced by more sedentary pursuits in past decades [5]. Physical activity, however, is favorably associated with physical, psychological/social, and cognitive health indicators of children 5–17 years old [11] while a decrease in physical fitness is associated with an increase in body mass index (BMI) among children and adolescents [12, 13].

In a cross-sectional study evaluating data of more than 4500 German children and adolescents aged 4–17 years, Woll et al. [14] found that overweight and obese children had lower values of physical fitness and gross motor coordination compared to their normal weight peers, and their physical limitations increased with increasing age through adolescence. Ruedl et al. [15] showed in a cross-sectional study including 304 Austrian primary school children that overweight and obesity was negatively associated with physical fitness. Results from a 2-year longitudinal study by D’Hondt et al. [16] including 754 primary school children from Belgium reveal the presence of a reciprocal causal relationship between children’s weight status and their level of gross motor coordination across development time, i.e., a lower performance in gross motor coordination at baseline translated into an increase in BMI z‑score over time while a higher baseline BMI z‑score predicted a decrease in subsequent performance of gross motor coordination [16]. In addition, Rodrigues et al. [17] tested in a 4-year longitudinal study among a cohort of 472 Portuguese primary school children how different developmental pathways of physical fitness relate to weight status (overweight and obesity) at the end of primary school. They found that children with a better (more rapid) increase in physical fitness through childhood are less prone to develop an overweight or obesity condition [17].

Longitudinal data of physical fitness and weight status are rare among Austrian primary school children. Primary schools are considered an ideal setting for implementing programs promoting enhanced physical activity and health behavior at an early age and addressing the majority of children [18]. Thus, the aim of this study was to evaluate the development of physical fitness of primary school children according to their weight status over a period of 2.5 years.

## Methods

Data from 488 primary school children from Tyrol in Austria were collected at 5 time points (from first to third grades) from 2014–2016 (every autumn and spring). This longitudinal study was performed according to the ethical standards of the 2008 Declaration of Helsinki and was approved by the educational board for Tyrol as well as by the Institutional Review Board for Ethical Issues of the University of Innsbruck. In addition, primary school directors agreed to participate and parents gave informed consent. Inclusion criterion for this study was children’s participation at all five test time points.

Children’s weight and height were measured in sports clothing and barefoot. Body weight was measured with a calibrated scale “Grundig PS 2010” (Grundig AG; Neu-Isenburg, Germany) with an accuracy of 0.1 kg and body height measures were taken using a mobile stadiometer “Seca 217” (Seca, Hamburg, Germany) with an accuracy of 0.1 cm. Based on these data, the BMI (kg/m^2^) was calculated for every child. According to the BMI reference system by Kromeyer-Hauschild et al. [19] children are regarded as being of normal weight if their weight was between the 10th and the 90th percentile. Values below the 3rd percentile and those between the 3rd and the 10th percentile were considered as being anorexic and underweight, respectively [19]. Values between the 90th and the 97th percentile and values above the 97th percentile were considered as being overweight and obese, respectively [19]. In line with our earlier work [15] children were classified according to their weight status into two groups: overweight (including overweight and obese children) and non-overweight (including anorexic, underweight and normal weight children). As the prevalence of overweight seems to increase during primary school time [17] we decided to use the weight data of the fifth test time point to differentiate between overweight and non-overweight children.

Physical fitness of primary school children was tested using the German motor performance test DMT 6–18 [20]. This standardized test battery consists of 8 items testing different subdomains of physical fitness [20]: 20 m sprint (sprint velocity), balancing backwards on three 3 m long beams with different widths (coordination in a task requiring precision), jumping sidewards over a middle line for 15 s (coordination under time pressure), stand-and-reach (flexibility), push-ups in a period of 40 s (strength endurance), sit-ups in a period of 40 s (strength endurance), standing long jump (power), and 6 min run (endurance). All tests were carried out by well-instructed physical education students in the sports halls at the participating schools following the exact instruction of the published test manual [20].

## Statistics

Data are presented as means and standard deviations and absolute and relative frequencies, respectively. Values of the eight test items were z‑transformed using the norming sample with analogous age and sex and a total z‑score was calculated as an indicator for children’s physical fitness according to Bös [20].

Using Shapiro-Wilk tests, total z‑scores and the z‑scores of all single test items were tested regarding a normal distribution of data. Independent t‑tests or Mann-Whitney U‑tests, as appropriate, were calculated to evaluate differences between overweight and non-overweight children with respect to physical fitness (total z‑scores and z‑scores of the 8 single items). Hedge’s g was used as effect size for group differences (small effect = 0.2; medium effect = 0.5, large effect = 0.8; [21]).

Repeated measurement analyses of variances (rANOVA) were calculated to evaluate differences in total z‑scores between the five test time points. These tests were carried out for overweight and non-overweight children and also with respect to gender. Partial η^2^ was used to quantify the effect size of the rANOVA (small effect = 0.02; medium effect = 0.13, large effect = 0.26; [21]). All *P*-values were two-tailed and values less than 0.05 were considered to indicate statistical significance.

## Results

A total of 266 children (55% boys) with a mean age of 6.4 ± 0.5 years at baseline and of 8.4 ± 0.5 years at the fifth time point met the inclusion criterion. Table 1 shows the mean BMI of the total cohort and relative frequencies of overweight children at all 5 test time points. The proportion of the overweight group increased over time from 11% at baseline to 22% at the fifth test time point.

**Table 1:** Body mass index of girls and boys as well as of the total cohort and percentage of overweight children at 5 test time points

	Autumn 2014	Spring 2015	Autumn 2015	Spring 2016	Autumn 2016
Body mass index of the total group (mean ± SD)	16.2 ± 2.0	16.3 ± 2.2	16.8 ± 2.5	16.8 ± 2.7	17.3 ± 2.8
Body mass index girls (mean ± SD)	16.1 ± 2.1	16.2 ± 2.0	16.7 ± 2.5	16.6 ± 2.8	17.2 ± 3.0
Body mass index boys (mean ± SD)	16.3 ± 1.9	16.5 ± 2.1	16.8 ± 2.4	17.0 ± 2.6	17.4 ± 2.7
Overweight children of the total group (%)	11.0	14.7	18.0	17.7	21.5
Overweight girls (%)	8.4	10.8	16.7	14.2	20.0
Overweight boys (%)	13.1	17.8	19.2	20.5	22.8

**Fig. 1:**
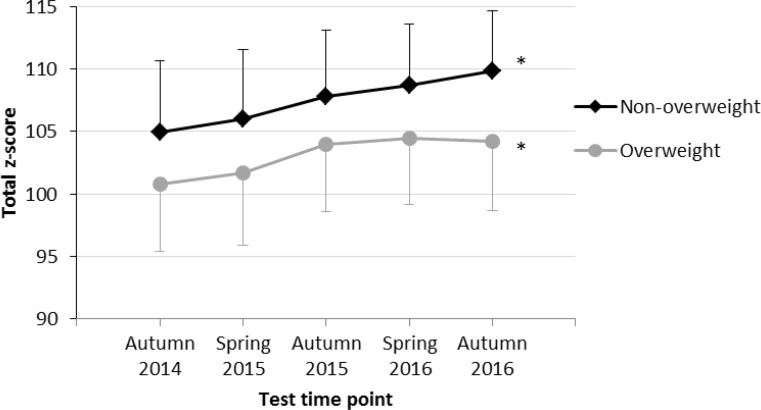
Physical fitness level (mean total z‑scores) at 5 test time points among overweight and non-overweight children. * indicates a significant increase in physical fitness according to results of rANOVA

Fig. 1 shows the mean total z‑scores of overweight and non-overweight children at the five test time points. According to the rANOVA, both overweight (F = 4.6; *p* < 0.001, partial η^2^ = 0.12) as well as non-overweight (F = 43.7; *p* < 0.001, partial η^2^ = 0.29) children showed a significant increase in physical fitness (total z‑scores) over a period of 2.5 years.

**Fig. 2:**
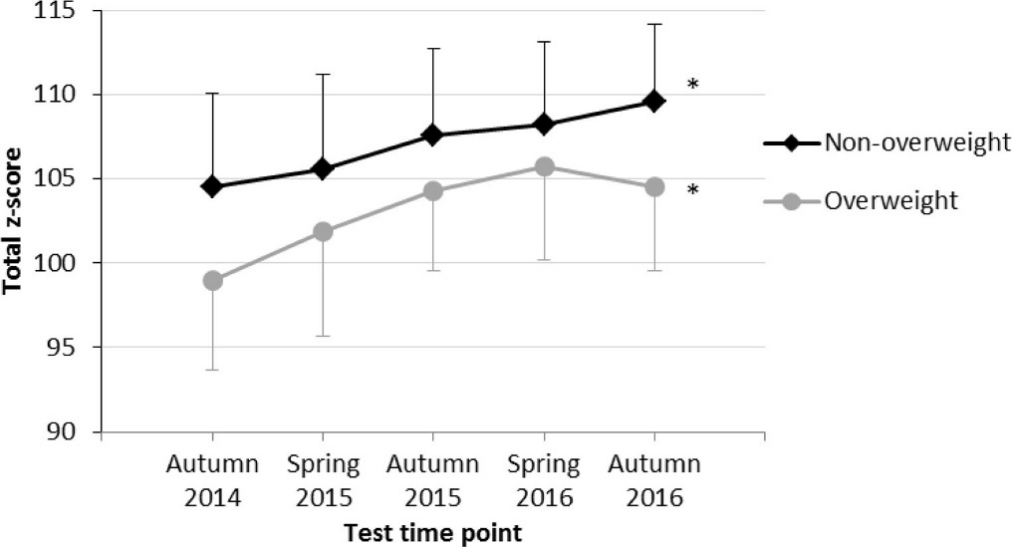
Physical fitness level (mean total z‑scores) at 5 test time points among overweight and non-overweight girls. * indicates a significant increase in physical fitness according to results of rANOVA

**Fig. 3:**
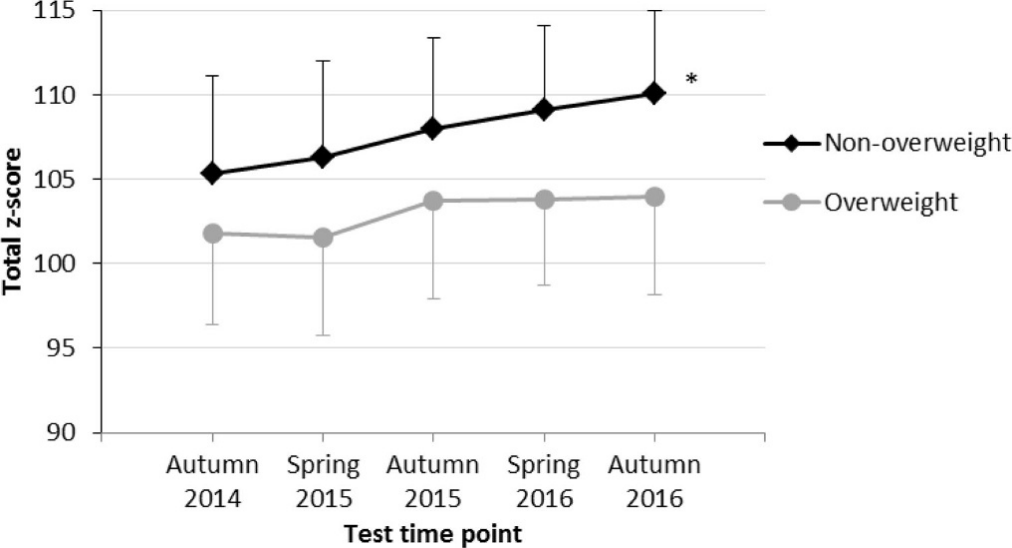
Physical fitness level (mean total z‑scores) at 5 test time points among overweight and non-overweight boys. * indicates a significant increase in physical fitness according to results of rANOVA

With respect to gender, physical fitness showed a significant increase in overweight (F = 5.8; *p* < 0.001, partial η^2^ = 0.20; Fig. 2) as well as in non-overweight (F = 37.4; *p* < 0.001, partial η^2^ = 0.28; Fig. 2) girls, and in non-overweight boys (F = 50.0; *p* < 0.001, partial η^2^ = 0.31; Fig. 3).

Table 2 shows total z‑scores and z‑scores of all 8 test items of overweight and non-overweight children. Non-overweight children showed significantly higher total z‑scores at all 5 time points as well as significantly higher mean z‑scores at all 5 time points within the tests 20m sprint, standing long jump and 6‑min run.

**Table 2:** Total z‑score and z scores of all test items of non-overweight and overweight children at 5 test time points

		Autumn 2014	Spring 2015	Autumn 2015	Spring 2016	Autumn 2016
Total z‑score	Non-overweight	104.9 ± 5.7	106.0 ± 5.6	107.8 ± 5.3	108.7 ± 4.9	109.9 ± 4.8
(Mean ± SD)	Overweight	100.8 ± 5.4	101.7 ± 5.8	104.0 ± 5.4	104.5 ± 5.3	104.2 ± 5.5
	*p*-value	<0.001	<0.001	<0.001	<0.001	<0.001
	Hedges‘ g	0.64	0.72	0.90	1.03	1.09
20-m sprint	Non-overweight	101.8 ± 9.0	104.4 ± 9.9	107.0 ± 7.4	108.6 ± 7.8	108.5 ± 8.2
(Mean z‑score ± SD)	Overweight	98.8 ± 8.8	101.5 ± 7.4	103.7 ± 9.9	104.9 ± 9.8	103.6 ± 8.5
	*p*-value	<0.001	<0.001	<0.001	<0.001	<0.001
	Hedges‘ g	0.31	0.43	0.58	0.43	0.56
Balancing backwards	Non-overweight	105.7 ± 10.7	106.2 ± 10.4	107.0 ± 9.4	109.3 ± 9.4	109.8 ± 8.6
(Mean z‑score ± SD)	Overweight	99.9 ± 9.2	101.3 ± 9.9	101.9 ± 8.8	105.8 ± 8.6	105.9 ± 10.1
	*p*-value	n.s.	0.002	<0.001	0.005	0.011
	Hedges‘ g	–	0.47	0.64	0.45	0.40
Jumping sidewards	Non-overweight	114.6 ± 10.9	116.6 ± 10.9	118.9 ± 9.7	121.4 ± 6.9	121.4 ± 5.6
(Mean z‑score ± SD)	Overweight	113.1 ± 10.0	111.9 ± 10.6	117.8 ± 8.3	120.5 ± 6.1	118.8 ± 7.4
	*p*-value	n.s.	0.002	<0.001	0.005	0.011
	Hedges‘ g	–	0.32	0.19	0.28	0.38
Stand-and-reach	Non-overweight	101.8 ± 9.6	102.9 ± 10.5	102.9 ± 9.9	102.2 ± 10.2	103.1 ± 10.7
(Mean z‑score ± SD)	Overweight	100.3 ± 8.4	103.9 ± 8.4	101.7 ± 9.8	98.6 ± 17.5	100.5 ± 9.4
	*p*-value	n.s.	n.s.	n.s.	n.s.	n.s.
	Hedges‘ g	–	–	–	–	–
Push-ups	Non-overweight	107.9 ± 10.6	111.4 ± 11.5	116.4 ± 9.5	119.4 ± 7.0	122.0 ± 5.9
(Mean z‑score ± SD)	Overweight	105.6 ± 9.8	109.0 ± 9.1	114.7 ± 8.6	117.0 ± 6.6	118.6 ± 7.9
	*p*-value	n.s.	n.s.	0.026	<0.001	0.001
	Hedges‘ g	–	–	0.31	0.55	0.49
Sit-ups	Non-overweight	99.3 ± 7.7	99.6 ± 6.8	101.5 ± 7.1	103.4 ± 7.1	103.9 ± 7.1
(Mean z‑score ± SD)	Overweight	95.8 ± 9.1	95.2 ± 8.9	97.3 ± 8.4	98.0 ± 8.2	98.0 ± 9.8
	*p*-value	n.s.	0.006	<0.001	<0.001	<0.001
	Hedges‘ g	–	0.48	0.77	0.87	0.75
Standing long jump	Non-overweight	102.7 ± 9.5	102.8 ± 9.4	103.1 ± 8.9	102.1 ± 9.3	104.6 ± 8.1
(Mean z‑score ± SD)	Overweight	98.8 ± 9.2	97.8 ± 9.9	97.7 ± 9.4	96.7 ± 9.4	95.5 ± 10.1
	*p*-value	<0.001	<0.001	<0.001	<0.001	<0.001
	Hedges‘ g	0.61	0.59	0.65	0.70	1.03
6-min run	Non-overweight	102.5 ± 12.0	103.0 ± 11.9	104.8 ± 10.0	101.1 ± 12.9	104.2 ± 11.4
(Mean z‑score ± SD)	Overweight	92.3 ± 10.9	91.2 ± 12.0	96.7 ± 8.8	91.1 ± 10.2	91.8 ± 11.1
	*p*-value	<0.001	<0.001	<0.001	<0.001	<0.001
	Hedges‘ g	0.86	0.88	0.99	0.93	1.08

## Discussion

The aim of the current study was to evaluate the development of physical fitness of overweight and non-overweight primary school children over a period of 2.5 years. The main findings were a significant increase of physical fitness over time among both overweight and non-overweight children; however, overweight children showed a significantly lower mean physical fitness level throughout the observation period compared to their non-overweight classmates. Our results reveal that the proportion of the overweight group doubled over the study period from 11% to 22%. In comparison, Rodrigues et al. [17] found in a 4-year longitudinal study of Portuguese primary school children an increase from children that were overweight or obese from 22.5% (first grade) to 30.0% (fourth grade). In addition, there is some evidence from cross-sectional studies conducted in Austria that the percentage of childhood overweight and obesity increases from preschool to the end of the primary school [15, 22, 23]. Greier et al. [22] reported among a cohort of Tyrolean preschool children with a mean age of 4.9 ± 0.5 years a prevalence of 7.6% overweight and of 5.5% obesity, while Ruedl et al. [15] found among a cohort of Tyrolean primary school children with a mean age of 8.0 ± 1.2 years a prevalence of 11.5% overweight and of 8.6% obesity. More recently, Furthner et al. [23] reported in 10-year-old children from Upper Austrian primary schools a prevalence of 20.7% overweight and of 6.0% obesity among boys and a prevalence of 18.3% overweight and of 5.5% obesity among girls. Causes for this increase of overweight and obesity during primary school might be a higher sedentary behavior during and after school hours, e. g. children spent up to half of their after school period with a sedentary behavior including homework, watching TV or other screen-based activities [24].

Our results show a significant increase of physical fitness among both overweight and non-overweight children; however, the effect for overweight children was small compared to a large-sized effect for non-overweight children indicating a more positive impact of physical education lessons at the primary schools for children of the latter group. There were two aspects regarding the time course of physical fitness of overweight children which were remarkable: 1) the time course started to plateau after the third test time point, and 2) overweight children did not even reach the mean baseline fitness level of non-overweight children over the entire study period (Fig. 1). These results might be explainable when comparing the increase in physical fitness of overweight and non-overweight children separately for girls (Fig. 2) and boys (Fig. 3). Whereas overweight and non-overweight girls as well as non-overweight boys showed a significant increase in physical fitness over time, no significant increase in physical fitness was found among overweight boys. As there is evidence that increased physical activity reduces the risk of becoming overweight or obese in adulthood [5], evidence-based preventive measures to reduce BMI and increase physical fitness in primary school children should especially focus on overweight and obese boys.

In general, our results reveal a significantly lower level of physical fitness among overweight children at all 5 time points and these differences remained constant over time (Fig. 1, Table 2). With respect to the results from the single test items (Table 2), overweight children showed significantly lower z‑scores at all 5 time points in the tests 20-m sprint, standing long jump and 6‑min run and significantly lower values in balancing backwards, jumping sideward and sit-ups at 4 time points while no significant difference was found within the stand-and-reach test between the two groups. These differences between overweight and non-overweight children are in line with previous research results [14, 15, 22] and might be partly caused by the fact that excessive body fat of overweight and obese children is an extra load to be moved during weight-bearing tasks [25]. Another cause for the lower fitness level of overweight children might be a lower self-esteem [26] and a lower motivation to participate in physical activity which is influenced by their perceived and actual physical competence as well as by their parents’ perceptions of their physical competence [27]. The observed gap of strength and endurance capacities between overweight compared to non-overweight children may contribute to the increased risk for cardiovascular diseases and orthopedic problems later on [6, 7] as Woll et al. [14] found that obese adolescents had upper body strength and power values that were comparable or lower than normal weight children 3 or more years younger.

There is evidence that daily lessons in physical education reduce adiposity and show a significantly lower rise in BMI during primary school as well as increase motor abilities and decrease daily sedentary activities [18, 28, 29]. In our previous work among primary school children [15], results of a multiple linear regression analysis revealed that more than 2 weekly lessons of physical education and sport club participation were associated with increased physical fitness among non-overweight (β = 0.22 and β = 0.16, respectively) children. Results were even more pronounced among overweight (β = 0.48 and β = 0.33, respectively) children [15]. In addition, Drenowatz et al. [30] showed that primary school children participating in organized sports more than once per week displayed higher physical fitness and were less likely to be overweight (odds ratio [OR]  = 0.52, *p* < 0.01). Thus, daily lessons in physical education and an increased participation in organized sports are strongly recommended to improve physical fitness especially among overweight primary school children.

In conclusion, overweight and non-overweight primary school children significantly increased their physical fitness over time; however, overweight children showed a significantly lower physical fitness level at all test time points that did not even achieve the mean baseline fitness level of non-overweight children. With respect to the increasing percentage of overweight children over the study period, evidence-based preventive measures to reduce overweight and increase physical fitness should be implemented at the earliest in primary schools, with a special focus on overweight boys.
